# Multiple sclerosis outcomes after cancer diagnosis in people with recorded chemotherapy exposure: A real-world MSBase study

**DOI:** 10.1177/13524585261460658

**Published:** 2026-08-02

**Authors:** Cassie Nesbitt, Paul Sanfilippo, Chao Zhu, Serkan Ozakbas, Alexandre Prat, Marc Girard, Pierre Duquette, Tomas Kalincik, Izanne Roos, Katherine Buzzard, Olga Skibina, Matteo Foschi, Andrea Surcinelli, Jeannette Lechner-Scott, Francesco Patti, Allan G Kermode, Marzena Fabis-Pedrini, William M Carroll, Suzanne Hodgkinson, Francois Grand’Maison, Eva Kubala Havrdova, Pavel Hradilek, Mario Habek, Davide Maimone, Raed Alroughani, Marta Vachova, Vincent van Pesch, Richard Macdonell, Daniele Spitaleri, Samia J Khoury, Oliver Gerlach, Koen de Gans, Pamela McCombe, Anneke Van Der Walt, Helmut Butzkueven, Vilija Jokubaitis

**Affiliations:** Department of Neuroscience, School of Translational Medicine, Monash University, Melbourne, VIC Australia; Department of Neurology, Alfred Hospital, Melbourne, VIC, Australia; Department of Neurology, University Hospital Geelong, Geelong, VIC, Australia; Department of Neuroscience, School of Translational Medicine, Monash University, Melbourne, VIC, Australia; Department of Neurology, Alfred Hospital, Melbourne, VIC, Australia; Department of Neuroscience, School of Translational Medicine, Monash University, Melbourne, VIC, Australia; Department of Neurology, Alfred Hospital, Melbourne, VIC, Australia; Medical Point Hospital, Izmir University of Economics, Izmir, Turkey; Multiple Sclerosis Research Association, Izmir, Turkey; CHUM and Universite de Montreal, Montreal, QC, Canada; CHUM and Universite de Montreal, Montreal, QC, Canada; CHUM and Universite de Montreal, Montreal, QC, Canada; Department of Neurology, Neuroimmunology Centre, The Royal Melbourne Hospital, Melbourne, VIC, Australia; Department of Medicine, CORe, The University of Melbourne, Melbourne, VIC, Australia; Department of Neurology, Neuroimmunology Centre, The Royal Melbourne Hospital, Melbourne, VIC, Australia; Department of Medicine, CORe, The University of Melbourne, Melbourne, VIC, Australia; Department of Neurosciences, Box Hill Hospital, Box Hill, VIC, Australia; Eastern Health Clinical School, Monash University, Box Hill, VIC, Australia; Department of Neuroscience, School of Translational Medicine, Monash University, Melbourne, VIC, Australia; Department of Neurology, Alfred Hospital, Melbourne, VIC, Australia; Department of Neurosciences, Box Hill Hospital, Box Hill, VIC, Australia; Santa Maria delle Croci Hospital, AUSL Romagna, Ravenna, Italy; Department of Biotechnological and Applied Clinical Sciences, University of L’Aquila, L’Aquila, Italy; Hunter Medical Research Institute, The University of Newcastle, Newcastle, NSW, Australia; Hunter New England Health, John Hunter Hospital, New Lambton Heights, NSW, Australia; Department GF Ingrassia Neurosciences, Università degli Studi di Catania, Catania, Italy; Multiple Sclerosis Unit, AOU Policlinico G Rodolico-San Marco, University of Catania, Catania, Italy; Perron Institute for Neurological and Translational Science, Sir Charles Gairdner Hospital, QEIIMC, University of Western Australia, Nedlands, WA, Australia; Personalised Medicine Centre, Health Futures Institute, Murdoch University, Perth, WA, Australia; Institute for Immunology and Infectious Diseases, Murdoch University, Perth, WA, Australia; Perron Institute for Neurological and Translational Science, Sir Charles Gairdner Hospital, QEIIMC, University of Western Australia, Nedlands, WA, Australia; Personalised Medicine Centre, Health Futures Institute, Murdoch University, Murdoch, WA, Australia; Perron Institute for Neurological and Translational Science, Sir Charles Gairdner Hospital, QEIIMC, University of Western Australia, Nedlands, WA, Australia; Department of Neurology, Faculty of Medicine, Liverpool Hospital, UNSW Sydney, Sydney, NSW, Australia; Department of Medicine, Immune Tolerance Laboratory, Ingham Institute, UNSW Sydney, Sydney, NSW, Australia; Neuro Rive-Sud, Greenfield Park, QC, Canada; Department of Neurology, First Faculty of Medicine, Charles University in Prague, Prague, Czechia; Department of Neurology, Faculty of Medicine, University of Ostrava, Ostrava, Czech Republic; Department of Neurology, Zagreb Medical School, Zagreb, Croatia; Division of Neurology, Garibaldi Hospital, Catania, Italy; Department of Medicine, Division of Neurology, Amiri Hospital, Sharq, Kuwait; Department of Neurology, Hospital Teplice of the Regional Health System, Teplice Hospital, Ústecký, Czechia; Department of Neurology, Cliniques Universitaires, Brussels, Belgium; Austin Hospital, Department of Neurology, Heidelberg, VIC, Australia; Department of Neurology, Azienda Ospedaliera di Rilievo Nazionale e di Alta Specialita San Giuseppe Moscati, Avellino, Italy; Nehme and Therese Tohme Multiple Sclerosis Center, American University of Beirut Medical Center, AUB, Beirut, Lebanon; Abu-Haidar Neuroscience Institute, American University of Beirut Medical Center, Beirut, Lebanon; Department of Neurology, Academic MS Center Zuyd, Zuyderland Medical Center, Sittard-Geleen, The Netherlands; Department of Neurology, School for Mental Health and Neuroscience, Maastricht University Medical Center, Maastricht, The Netherlands; Department of Neurology, Groene Hart Ziekenhuis, Gouda, The Netherlands; Department of Neurology, Royal Brisbane Hospital, Brisbane, OLD, Australia; The University of Queensland, Brisbane, QLD, Australia; Department of Neuroscience, School of Translational Medicine, Monash University, Melbourne, VIC, Australia; Department of Neurology, Alfred Hospital, Melbourne, VIC, Australia; Department of Neuroscience, School of Translational Medicine, Monash University, Melbourne, VIC, Australia; Department of Neurology, Alfred Hospital, Melbourne, VIC, Australia; Department of Neuroscience, School of Translational Medicine, Monash University, Melbourne, VIC, Australia; Department of Neurology, Alfred Hospital, Melbourne, VIC, Australia

**Keywords:** Multiple sclerosis, chemotherapy, age, relapse, cancer

## Abstract

**Background and objective:**

As more people with multiple sclerosis (MS) survive cancer, questions about MS management after cancer are increasingly relevant. This study aimed to describe post-cancer treatment patterns and MS outcomes in people with recorded chemotherapy exposure.

**Methods:**

Using MSBase, we identified people with MS with cancer and chemotherapy exposure. Time to first relapse and 6-month confirmed disability progression (CDP) were analysed using Cox models with disease-modifying therapy (DMT) as a time-varying covariate. Outcomes were contextualised using 1:2 propensity-matched MS controls without cancer or chemotherapy.

**Results:**

In total, 363 individuals were followed for 2.8 years (median) after cancer. Older age at cancer was associated with lower relapse hazard (hazard ratio (HR) = 0.96 per year, *p* = 0.008), while DMT category was not. The DMT category was not associated with CDP. In matched analysis (256 vs. 505), relapse hazard was lower during the first year after cancer (HR = 0.30, *p* = 0.015) with no difference thereafter; CDP risk was similar (HR = 1.13, *p* = 0.60).

**Conclusion:**

Post-cancer DMT category was not associated with relapse or CDP. Lower first-year relapse hazard, together with lower relapse hazard at older age, may provide cautious reassurance regarding early post-cancer inflammatory activity in similar clinical contexts, although the drivers of the first-year signal remain uncertain.

## Introduction

Coexisting multiple sclerosis (MS) and malignancy is increasingly encountered in neurology care, yet evidence to guide MS treatment when a person with MS (pwMS) receives systemic cancer chemotherapy remains limited.^1^ MS disease-modifying therapy (DMT) aims to reduce inflammatory relapse activity and disability accumulation by modulating immune function.^1^ Cancer chemotherapy uses cytotoxic regimens that commonly cause neutropenia and lymphopenia, with variable recovery.^1,2^ In this dual diagnosis setting, clinicians must decide whether to continue, pause, switch or restart DMT during and after cancer treatment, with limited data to guide these decisions.

Certain cytotoxic agents, including cyclophosphamide and mitoxantrone, have demonstrated efficacy in MS, and autologous haematopoietic stem cell transplantation (AHSCT) shows that a defined course of intensive chemotherapy can produce sustained suppression of MS inflammatory activity.^1,3,4^ However, routine oncological regimens differ in agent, intensity, schedule and intent, and their effects on MS outcomes remain uncertain.^1^

The 2023 French MS Society recommendations provide expert consensus guidelines for managing MS in the context of cancer.^4^ They emphasise interdisciplinary collaboration, consideration of pausing DMT during lymphoablative therapies and reinitiation guided by individual clinical assessment.^4^ Empirical outcome data are needed to inform these recommendations. Using MSBase, we addressed two questions: (1) What real-world patterns of DMT use are observed after cancer diagnosis in pwMS with recorded systemic chemotherapy exposure? and (2) What MS outcomes are observed after cancer diagnosis in this cohort?

## Methods

### Standard protocol approvals, registrations and patient consents

MSBase is registered with the World Health Organization International Clinical Trials Registry Platform (ID ACTRN12605000455662). Ethics approval or exemption was obtained from each participating site’s institutional review board, including approval from the Alfred Health Human Research Ethics. Written informed consent was obtained from all enrolled participants.

This study is reported in accordance with the Strengthening the Reporting of Observational Studies in Epidemiology (STROBE) guidelines.^5^

### Study design and population

This retrospective multicentre cohort study analysed prospectively collected data from the MSBase registry.

**Figure 1.**
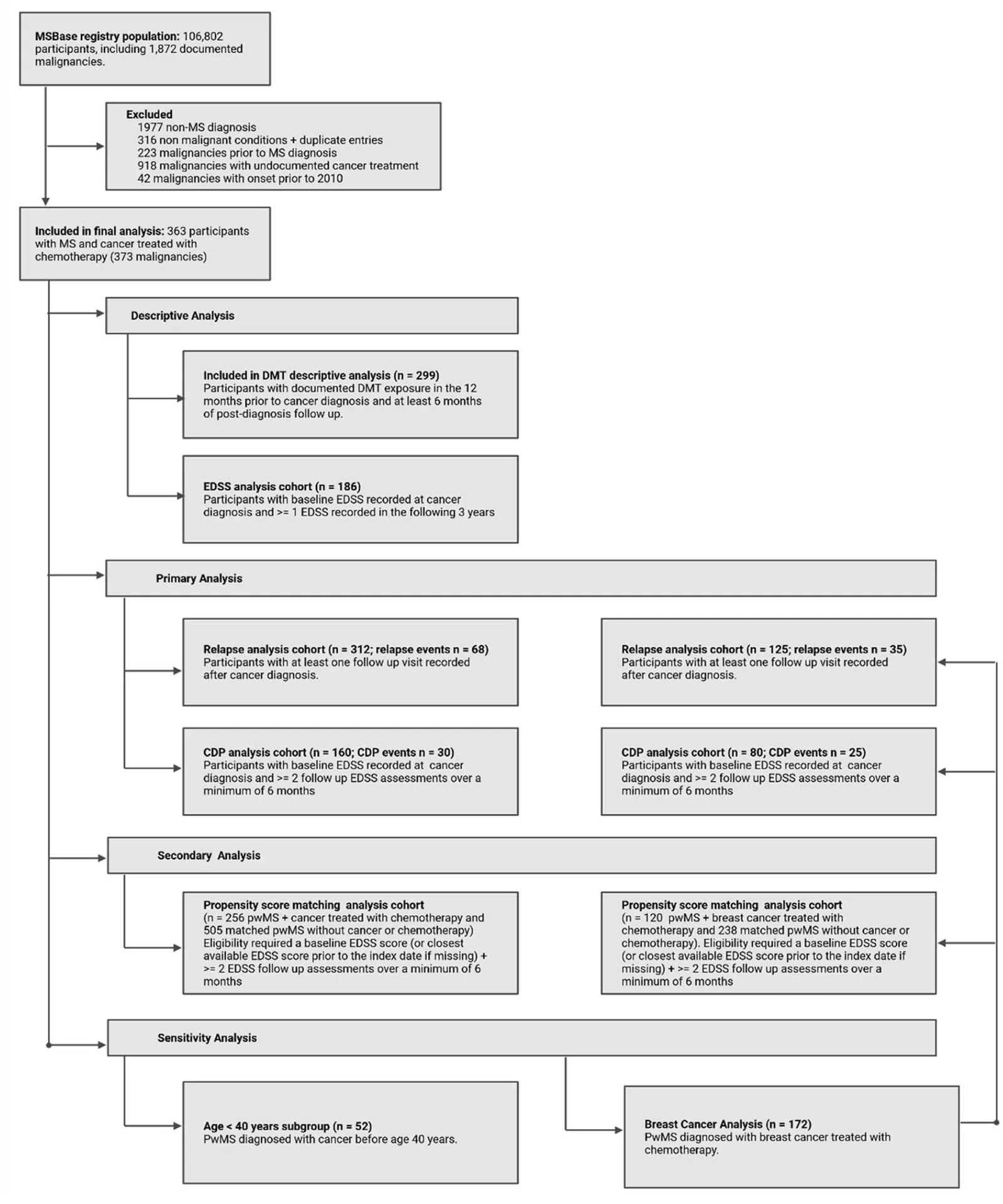
Flow diagram of cohort selection and analytic populations from the MSBase registry. PwMS: people with multiple sclerosis; DMT: disease-modifying therapy; EDSS: Expanded Disability Status Scale; CDP: confirmed disability progression.

Inclusion criteria comprised a confirmed MS diagnosis, a malignancy occurring after MS onset and a clinician-recorded exposure to systemic cancer chemotherapy (Figure 1). Malignancies diagnosed before 2010, before the introduction of high-efficacy DMTs, were excluded. Analyses for individuals with multiple malignancies used the first cancer diagnosis.

Chemotherapy exposure was recorded as a binary variable; in a verified subset (*n* = 41), this aligned with recognised cytotoxic regimens including anthracyclines, taxanes, platinum agents, antimetabolites, alkylating agents and combination protocols.

All analyses used complete case data without imputation. Data were extracted on 1 March 2024.

### Statistical analysis

Analyses were conducted using R (version 4.3.3). Continuous variables were summarised as means with standard deviations or medians with interquartile ranges (IQRs), and categorical variables as frequencies and percentages. Group comparisons between post-cancer DMT categories used the Kruskal–Wallis test for continuous variables and the chi-square test for categorical variables.

MS was classified as relapsing (clinically isolated syndrome and relapse-onset MS) or progressive (primary progressive, secondary progressive and progressive relapsing MS) at cancer diagnosis.

Relapse was defined as new or recurrent neurological symptoms lasting at least 24 hours, occurring in the absence of fever, infection or other external factors, as documented by the treating clinician. MRI confirmation is not required.

Confirmed disability progression (CDP) was defined as an increase in Expanded Disability Status Scale (EDSS) from baseline of at least 1.0 point, or at least 0.5 points where baseline EDSS was 5.5 or higher, with progression confirmed at a subsequent EDSS assessment recorded a minimum of 6 months after baseline. Time to CDP was measured from the date of cancer diagnosis to the first confirmed progression event. Index EDSS differed by analysis, using the EDSS closest to cancer diagnosis within 6 months for descriptive EDSS change windows, the closest value within the prior 12 months for adjusted primary models and EDSS at the index visit or the closest prior value where missing for propensity score matching. Participants without CDP were censored at the date of their last available EDSS assessment.

For time-to-event analyses, follow-up commenced at the date of cancer diagnosis, as chemotherapy timing and related immune recovery data were unavailable. CDP analyses were restricted to individuals with three EDSS assessments recorded from cancer diagnosis onwards, with a minimum interval of 6 months between consecutive assessments. Relapse analyses included all individuals with at least one post-diagnosis follow-up visit.

#### Primary analysis

Time to first relapse and 6-month CDP were assessed using Cox proportional hazards models (complete case analysis). Models were adjusted for age at cancer diagnosis, disease duration, MS phenotype, EDSS at cancer diagnosis, sex, relapse count in the 12 months prior and DMT modelled as a time-varying exposure reflecting treatment status at the time of the event. DMTs were categorised *a priori* by author consensus as immunosuppressing (CD20 depleting therapies (anti-CD20), sphingosine 1 phosphate (S1P) modulators, Bruton’s tyrosine kinase inhibitors (BTKIs), natalizumab, daclizumab, alemtuzumab, fumarates, cladribine, cyclophosphamide, mitoxantrone and AHSCT), or non-immunosuppressing (teriflunomide, glatiramer acetate and interferons) to reflect overall immunosuppressive burden in the context of cancer and chemotherapy, rather than an efficacy hierarchy.

Schoenfeld residuals were used to assess proportional hazards assumptions.

#### Secondary analysis

Propensity score matching (PSM) compared pwMS with cancer and chemotherapy to pwMS without cancer or chemotherapy to contextualise MS outcomes after cancer. Both groups were required to have complete baseline covariates and sufficient follow-up. Matching was 1:2 using nearest neighbour matching with replacement at the index visit level, with exact matching on age at cancer or index visit and visit calendar year. The propensity score used a 0.05 calliper and included relapse count in the 12 months pre-index, EDSS at index, age at MS onset, disease duration, sex, current DMT class at index and prior DMT exposure (binary indicators by pharmacological class). Balance was assessed using absolute standardised mean differences.

Time to first relapse and time to CDP were analysed using Cox models with robust standard errors clustered by participant to account for matching with replacement. Follow-up commenced at cancer diagnosis or the matched index visit and ended at the first event or the last recorded visit. MS phenotype at index was included in all models, and any covariates remaining imbalanced after matching were added as adjustment terms. Proportional hazards were assessed, and where violated, a piecewise Cox model with yearly intervals was used for interpretability and alignment with standard annual survivorship reporting.

Post-cancer DMT was not modelled as a time-varying covariate because post-cancer prescribing is shaped by the cancer treatment pathway and clinical recovery, and adjustment would risk exposure-induced confounding by conditioning on a mediator or collider. The matched analysis, therefore, reflects the broader cancer treatment pathway in pwMS with recorded chemotherapy exposure and cannot isolate the contribution of chemotherapy from other components of post-cancer care.

#### Sensitivity analyses

A sensitivity analysis replicating both the primary and secondary analyses was conducted in the breast cancer subgroup. Breast cancer was the most common malignancy in the cohort and was selected due to its relatively standardised treatment protocols^6^ and sufficient sample size.

A second sensitivity analysis descriptively compared outcomes in a younger subgroup (age at cancer <40) to those aged >40 years.

## Results

### Participant characteristics

We assessed 373 malignancies in 363 people with MS (pwMS) with recorded chemotherapy exposure as part of their cancer management (Figure 1). Study participants were from 77 centres across 23 countries (Table 1). The median calendar year of cancer diagnosis was 2017 (range: 2010–2024). Breast cancer was most common (46.2%), reflecting both its global prevalence in 2024^7^ and the predominance of women in the MS population (79.4% in our cohort). Median age at cancer diagnosis was 60.7 years, with a median interval of 13.6 years from MS onset. Cancer outcomes were available for 279 individuals, mostly reflecting status at data entry: 137 (49.1%) had ongoing cancer, 122 (43.7%) were in remission, and 15 (5.4%) had died.

**Table 1.** Demographic and clinical characteristics of the study sample.

Characteristic	Value		
Sex^a^	Female: 288 (79.4%)		
	Male: 75 (20.6%)		
**MS characteristics**			
Median age at MS onset	37.01 years (IQR: 15.4)		
MS phenotype at cancer diagnosis	Clinically isolated syndrome: 22 (5.9%)		
	Relapse-onset MS: 270 (72.4%)		
	Primary progressive: 17 (4.6%)		
	Progressive relapsing: 11 (2.9%)		
	Secondary progressive: 53 (14.2%)		
MS duration with cancer	13.6 years (IQR: 15.2)		
Relapse count in the 12 months before cancer	Median 0 (IQR: 0–0); 38/363 (10.5%) had at least one recorded relapse		
**Disability outcomes (EDSS)**			
Timepoint (±6 months)	Median (IQR)	Missing (%)	
Baseline for cancer	2.5 (3.375)	34.4	
Year 1 post-cancer	3 (4)	41.9	
Year 2 post-cancer	3.5 (4.0)	65.6	
Year 3 post-cancer	3.0 (4.0)	58.4	
Year 4+ post-cancer	3.0 (4)	66.4	
**Cancer characteristics**			
Median age at cancer	60.7 years (IQR: 15.7)		
Follow-up time after cancer	Median: 2.8 years (IQR: 5.4)		
	58/363 (16.0%) were lost to follow-up by 6 months		
Cancer outcomes when recorded, *n* = 279 *N* (%)	Deaths: 15 (5.4%)		
	Ongoing cancer: 137 (49.1%)		
	Remission: 122 (43.7%)		
	Not specified: 5 (1.8%)		
**Cancer types (%)**			
Breast: 46.2%	Blood: 12.0%	Colo-rectal: 11.5%	Lung: 4.3%
Ovarian: 3.5%	Gastro-oesophageal: 2.4%	Uterus: 1.9%	Head and neck: 1.9%
Pancreas: 1.9%	Hepatobiliary: 1.6%	Melanoma: 1.6%	Cervix: 1.3%
Adreno-urinary: 1.3%	Testicular: 1.3%	Brain and nerve: 1.1%	Prostate: 1.1%
Sarcoma: 0.8%	CNS lymphoma: 0.8%	Skin: 0.8%	Mesothelioma: 0.5%
Unknown primary: 0.5%	Anal: 0.5%	Thyroid: 0.3%	Neuroendocrine: 0.3%
Primary peritoneal: 0.3%	Vulvovaginal: 0.3%		
**Geographical distribution (%)**			
Argentina: 1.1%	Australia: 25.9%	Belgium: 2.2%	Brazil: 0.28%
Canada: 7.99%	Colombia: 0.28%	Czech Republic: 16%	Croatia: 1.93%
Egypt: 0.55%	Iran: 1.38%	Italy: 12.4%	Kuwait: 1.38%
Lebanon: 1.38%	Netherlands: 3.03%	Portugal: 0.83%	Romania: 0.28%
Saudi Arabia: 0.28%	Slovenia: 0.28%	Spain: 6.06%	Tunisia: 0.83%
Turkey: 14.6%	United Kingdom: 0.83%	United States of America: 0.28%	

a Sex is reported using the binary gender variable captured in MSBase.

### Changes in EDSS scores post-cancer diagnosis

**Figure 2.**
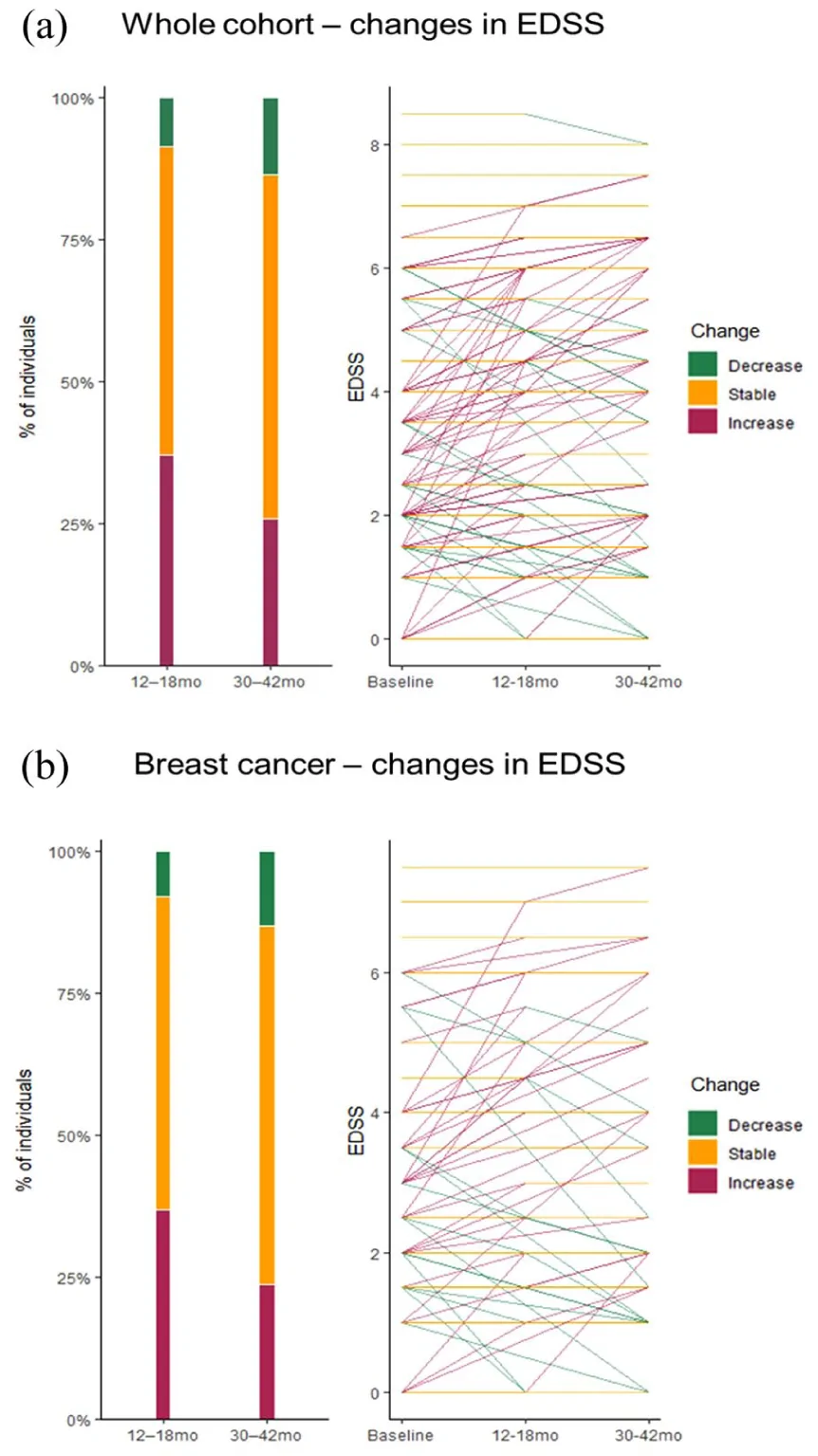
Panel (a) shows the full cohort (*n* = 186), and panel (b) shows the breast cancer subgroup (*n* = 88). Stacked bar plots show the proportion of individuals whose EDSS decreased, remained stable or increased between baseline and the 12-to-18-month and 30-to-42-month windows, with adjacent spaghetti plots illustrating individual trajectories. Only individuals with EDSS recorded at baseline and at least one follow-up were included. Baseline EDSS was defined as the score closest to cancer diagnosis within 6 months, and follow-up scores were the first available within each window. Among individuals with available EDSS, the median EDSS was 2.5 at baseline, 3.4 at 12 months and 3.0 at 24 months in the full cohort. In the breast cancer subgroup, median EDSS remained 2.5 across windows, with interquartile ranges of 3.25 at baseline and 3.5 at 12 and 24 months.

There was a high rate of missing EDSS data in the early follow-up period after cancer. Among available data, median EDSS increased from 2.5 at the time of cancer diagnosis to 3.4 at 12–18 months and then decreased to a median of 3.0 from 24 months onwards (Table 1 and Figure 2).

### DMT use after cancer diagnosis

**Figure 3.**
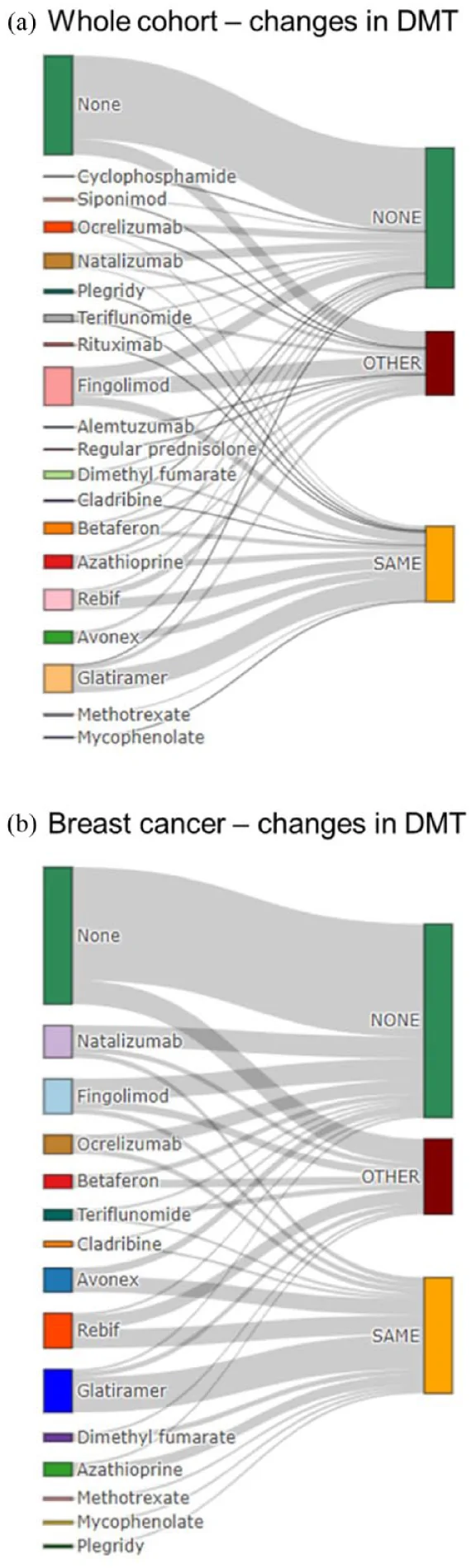
Disease-modifying therapy use at cancer diagnosis and 12 months after diagnosis. Sankey diagrams show DMT status at cancer diagnosis (left) and at 12 months after diagnosis (right). Each ribbon represents individuals and shows whether they remained on the same agent, switched to a different agent or DMT class, stopped DMT or initiated DMT from no recorded DMT. Panel (a) includes the full cohort (*n* = 299). At cancer diagnosis, 34.8% (*n* = 104) were receiving immunosuppressing DMTs, 29.8% (*n* = 89) were receiving non-immunosuppressing DMTs, and 35.5% (*n* = 106) had no recorded DMT. At 12 months, 19.7% (*n* = 59) were receiving immunosuppressing DMTs, 31.1% (*n* = 93) were receiving non-immunosuppressing DMTs, and 49.2% (*n* = 147) had no recorded DMT. Agent-level counts at each time point are reported in Supplemental eTable 1. Definitions of DMT classes are provided in “Methods” section. Panel (b) includes the breast cancer subgroup (*n* = 138).

A 12-month timeframe (±6 months) was selected to evaluate DMT use following cancer diagnosis, allowing time for treatment completion, management of complications and clinical reassessment. DMT exposure shifted over this period (Figure 3). At diagnosis, 35.5% (*n* = 106) had no recorded DMT, 29.8% (*n* = 89) were receiving non-immunosuppressing DMTs, and 34.8% (*n* = 104) were receiving immunosuppressing DMTs; by 12 months, 49.2% (*n* = 147) had no recorded DMT, 31.1% (*n* = 93) were receiving non-immunosuppressing DMTs, and 19.7% (*n* = 59) were receiving immunosuppressing DMTs. Among those receiving an immunosuppressing DMT at diagnosis, 39.4% remained on an immunosuppressing DMT, 17.3% switched to a non-immunosuppressing DMT, and 43.3% had no recorded DMT at 12 months. Among those receiving a non-immunosuppressing DMT at diagnosis, 77.5% remained on treatment, and 22.5% had no recorded DMT at 12 months. Among those with no recorded DMT at diagnosis, 22.6% had initiated treatment by 12 months (Figure 3, Supplemental eTables 1 and 2).

At 12 months, those receiving non-immunosuppressing DMTs were younger than those with no recorded DMT, and baseline EDSS was higher in the immunosuppressing than non-immunosuppressing group; no other differences were observed (Supplemental eTable 1).

### Primary analysis: MS outcomes after cancer diagnosis in pwMS and recorded chemotherapy

The relapse analysis included 312 participants with a median follow-up of 2.8 years after cancer diagnosis; 68 (21.8%) experienced a relapse over the observation window. In adjusted Cox models, older age at cancer diagnosis was associated with lower relapse hazard (hazard ratio (HR) = 0.96 per year, 95% confidence interval (CI) = 0.93–0.99, *p* = 0.008), while relapsing phenotype was associated with higher relapse hazard (HR = 4.31, 95% CI = 1.56–11.87, *p* = 0.005). There was no evidence that the current DMT category at the time of relapse was associated with relapse hazard for non-immunosuppressing DMT exposure (HR = 1.74, 95% CI = 0.86–3.50, *p* = 0.13) or immunosuppressing DMT exposure (HR = 1.19, 95% CI = 0.46–3.05, *p* = 0.72).

Relapse incidence rates by time-varying DMT exposure were 7.24 per 100 person years off treatment (56 events over 773 person years), 12.8 per 100 person years during non-immunosuppressing DMT exposure (9 events over 70.4 person years) and 7.86 per 100 person years during immunosuppressing DMT exposure (3 events over 38.2 person years).

In a descriptive subgroup of pwMS receiving a DMT at cancer diagnosis who discontinued and remained off treatment at 12 months (*n* = 39), 7 (18.0%) experienced a first relapse; all occurred beyond 12 months after cancer diagnosis, with the median time to relapse of 3.69 years (IQR: 1.87–4.0).

The CDP analysis included 160 participants: 30 experienced CDP. Neither non-immunosuppressing DMTs (HR = 0.93, 95% CI = 0.44–1.97, *p* = 0.85) nor immunosuppressing DMTs (HR = 1.02, 95% CI = 0.47–2.21, *p* = 0.97) were associated with CDP risk. Higher EDSS (HR = 1.19, 95% CI = 1.01–1.41, *p* = 0.041) and progressive phenotype (HR = 2.42, 95% CI = 1.05–5.56, *p* = 0.038) were associated with a higher progression risk.

### Secondary analysis: contextualising MS outcomes after cancer diagnosis

**Figure 4.**
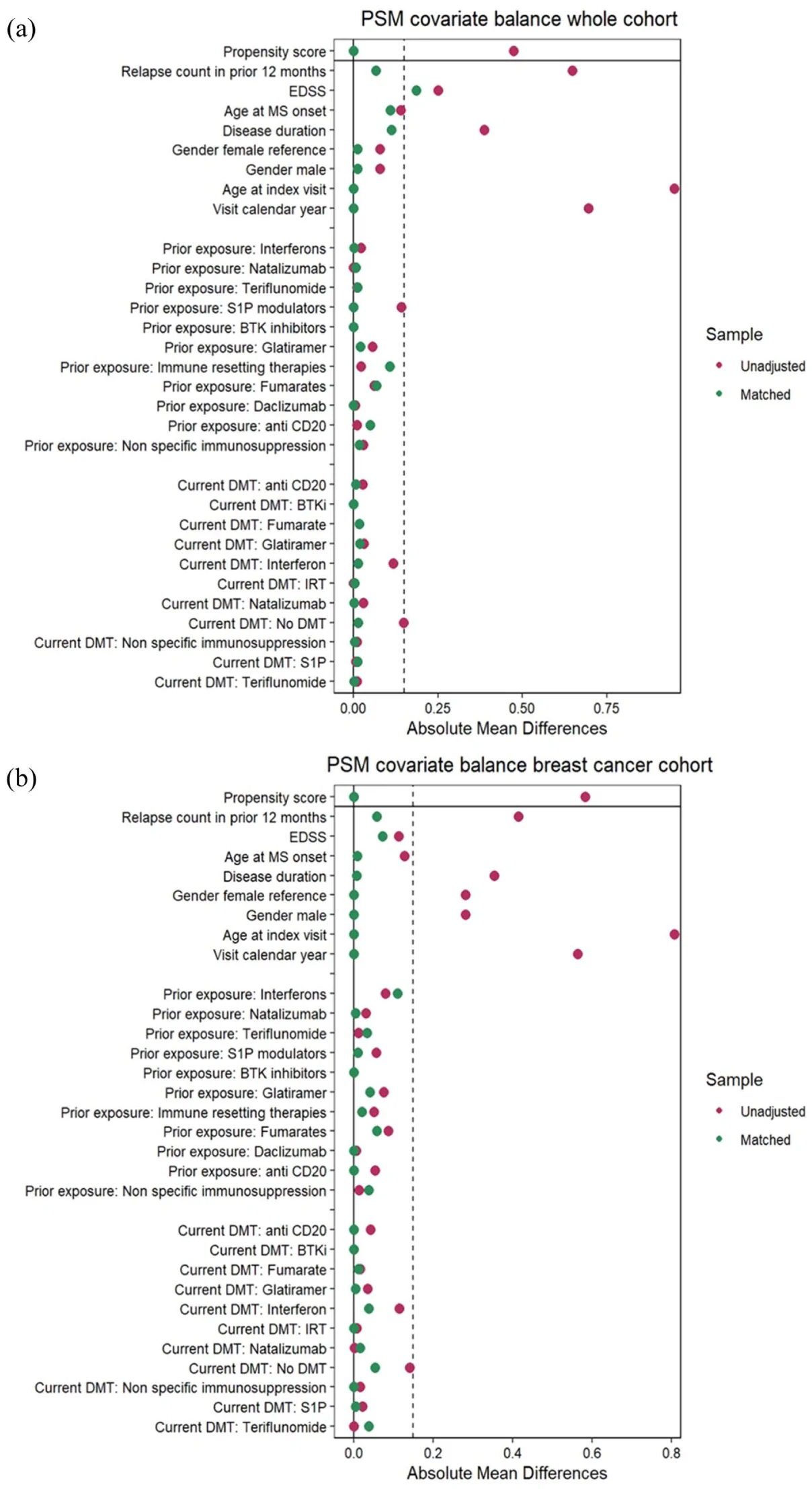
Covariate balance before and after propensity score matching in the full cancer cohort and breast cancer subgroup. Love plots show absolute standardised mean differences for baseline covariates in the whole cohort (a) and breast cancer subgroup (b). Maroon points indicate unadjusted samples, and green points indicate matched samples. The dashed line marks the balance threshold of 0.15. Most covariates achieved acceptable balance after matching. EDSS remained above the balance threshold after matching in the whole cohort (Panel (a)), with a residual difference of 0.29 EDSS units (3.21 vs. 3.50). EDSS and MS phenotype at index were included as covariates in matched outcome models. PSM: propensity score matching; EDSS: Expanded Disability Status Scale. Combined interferons: Interferon beta 1a, interferon beta 1b, pegylated interferon beta 1 and interferon therapy not otherwise specified. Fumarates: dimethyl fumarate and diroximel fumarate. S1P: fingolimod, siponimod, ozanimod and ponesimod. BTKI: Bruton’s tyrosine kinase inhibitors. Anti-CD20: rituximab, ocrelizumab and ofatumumab. Combined IRT: cladribine and alemtuzumab. Non-specific immunosuppression: mycophenolate, methotrexate and azathioprine.

**Figure 5.**
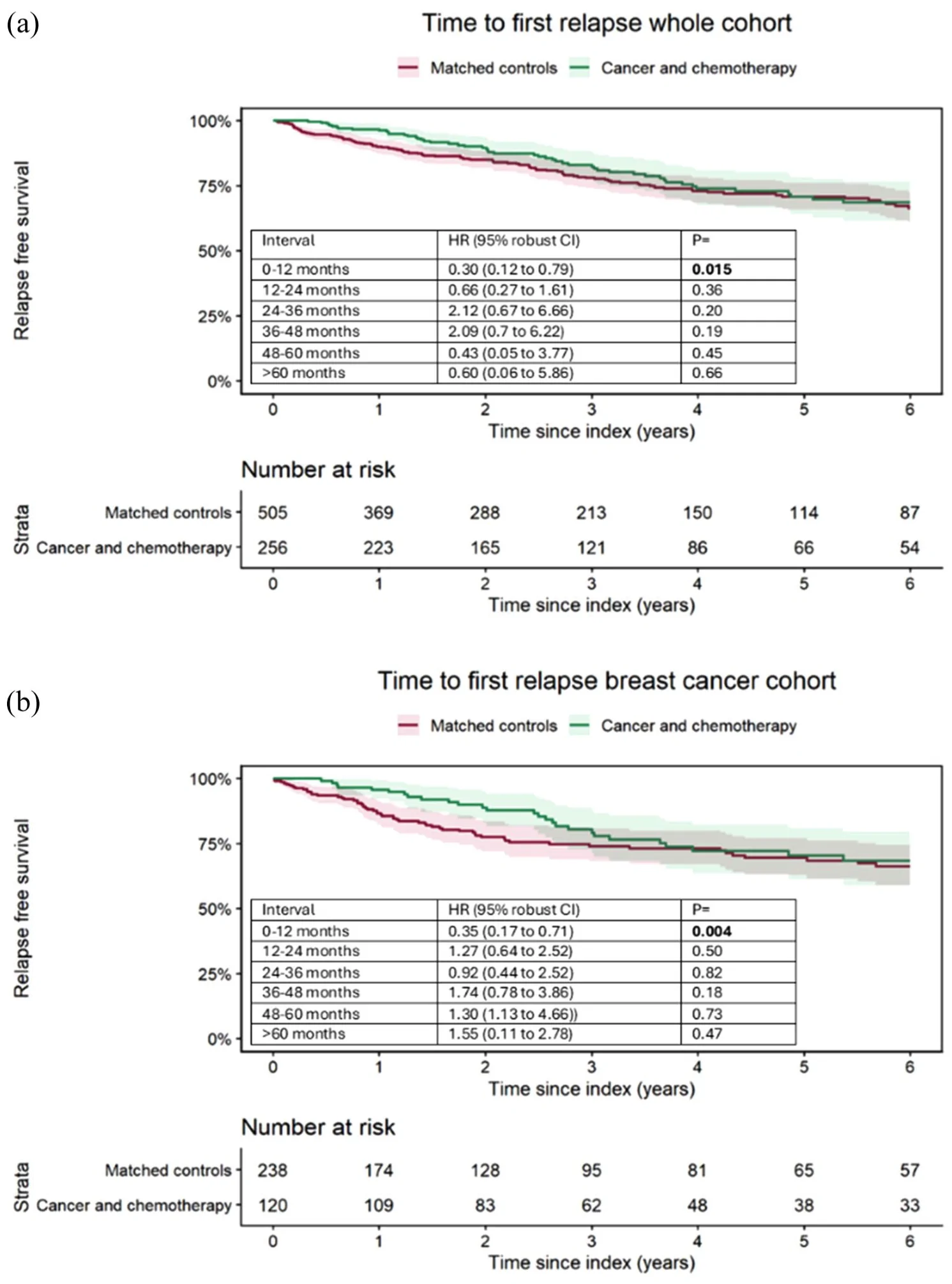
Time to first relapse since index in propensity score matched analyses. Panel (a) shows the whole cohort (256 people with multiple sclerosis and cancer treated with chemotherapy and 505 matched controls). Panel (b) shows the breast cancer subgroup (120 people with multiple sclerosis and cancer treated with chemotherapy and 238 matched controls). Curves show relapse-free survival over time, with shaded 95% confidence intervals. Time since index is measured from the date of cancer diagnosis in the cancer and chemotherapy group and from the matched index visit in controls. Tables report interval-specific hazard ratios with 95% robust confidence intervals and *p* values from piecewise Cox proportional hazards models comparing the cancer and chemotherapy group with matched controls, using 12-month intervals with a final interval of more than 60 months. Numbers at risk are shown below each panel. HR: hazard ratio; CI: confidence interval.

The matched analysis included 256 pwMS with cancer and chemotherapy and 505 matched MS controls without cancer or chemotherapy exposure (Figure 4(a), Supplemental eTable 3). Relapse hazard was lower in the cancer and chemotherapy group in year 1 (HR = 0.30, 95% robust CI = 0.12–0.79, *p* = 0.015) with no difference thereafter (Figure 5(a)). Relapsing phenotype was associated with higher relapse hazard (HR = 2.27, 95% robust = 1.28–4.03, *p* = 0.005); EDSS was not (HR = 0.99, 95% robust CI = 0.90–1.08, *p* = 0.76). CDP risk did not differ between groups (HR = 1.13, 95% robust = 0.71–1.79, *p* = 0.60). Higher EDSS was associated with higher CDP risk (HR = 1.10, 95% robust CI = 1.01–1.20, *p* = 0.035); progressive phenotype was not (HR = 1.33, 95% robust CI = 0.86–2.07, *p* = 0.20).

### Sensitivity analysis: breast cancer subgroup

There were 172 breast cancers among 167 individuals (Supplemental eTable 4). Median age at MS onset was 35.9 years, and median age at cancer diagnosis was 48.6 years, compared with 55.3 years in those diagnosed with non-breast cancers (*p* < 0.001). Most had relapse-onset MS (73.4%). EDSS trajectories were variable but broadly aligned with the full cohort; the median EDSS score remained stable at 2.5 despite individual fluctuation (Figure 2(b), Supplemental eTable 4). Post-cancer, DMT exposure again showed a shift towards treatment discontinuation or non-immunosuppressing therapies (Figure 3(b)).

Following cancer diagnosis, relapse risk was greater in those with relapsing (HR = 4.67, 95% CI = 1.16–18.70, *p* = 0.03) rather than progressive MS, while older age at cancer diagnosis was associated with lower relapse risk (HR = 0.94, 95% CI = 0.91–0.99, *p* = 0.025). Post-cancer use of non-immunosuppressing (HR = 1.75, 95% = 0.46–6.50, *p* = 0.40) and immunosuppressing (HR = 1.50, 95% CI = 0.42–5.32, *p* = 0.53) DMTs was not associated with relapse risk or CDP (non-immunosuppressing HR = 1.53, 95% CI = 0.54–4.33, *p* = 0.43; immunosuppressing HR = 1.46, 95% CI = 0.53–3.99, *p* = 0.46).

In the breast cancer subgroup, PSM analyses (Supplemental eTable 5, Figure 4(b)) showed a lower relapse hazard in the cancer chemotherapy group than matched controls during the first year after index (HR = 0.35, 95% robust CI = 0.17–0.71, *p* = 0.004; Figure 5(b)), with no difference thereafter and no difference in CDP (HR = 1.13, 95% robust CI = 0.69–1.79, *p* = 0.60).

### Subgroup analysis of individuals under 40

To examine the impact of age, a subgroup analysis was conducted in individuals diagnosed with cancer before age of 40 years (*n* = 55; median age at diagnosis: 36.3 years, IQR: 5.7; Supplemental eTable 6). Among those with follow-up, relapse occurred in 30.3% of individuals under 40 years, compared with 18.5% of those aged 40 years or older (odds ratio (OR) = 1.90, 95% CI = 0.72–4.74, *p* = 0.15; Fisher’s exact test).

## Discussion

This MSBase analysis provides real-world data on post-cancer DMT patterns and MS outcomes in pwMS with recorded chemotherapy exposure. Although not designed to evaluate the 2023 French guidelines,^4^ it adds observational data to an area where management remains guided largely by expert consensus.

*Question 1: What post-cancer DMT patterns were observed in real-world practice?*
  By 12 months after cancer diagnosis, no recorded DMT was more common, and immunosuppressing DMT exposure was less common than at diagnosis, with discontinuation, switching and some treatment initiation observed. These data describe post-cancer prescribing patterns rather than treatment intent, but are broadly consistent with recommendations for reassessment of MS therapy in the context of cancer.^4^
*Question 2: What MS outcomes were observed after cancer diagnosis in this cohort?*
  Post-cancer DMT category was not associated with relapse hazard in either the primary cohort or the breast cancer subgroup. Within the primary cohort, relapse hazard was higher in those with relapsing phenotypes and lower with older age at cancer diagnosis. Relapse was more frequent in those diagnosed before age of 40 years, consistent with known age-related patterns of inflammatory disease activity.^8^

To contextualise relapse activity, we compared pwMS with cancer and recorded chemotherapy exposure with propensity-matched MS controls without cancer or chemotherapy. This was a pragmatic rather than causal comparison, because the post-cancer treatment pathway and subsequent MS management could not be replicated in cancer-free controls. In matched analyses, relapse hazard was lower during the first year, with no difference thereafter. Of note, relapse ascertainment in our cohort was restricted to clinician-recorded activity without MRI confirmation. Therefore, in pwMS with cancer and recorded chemotherapy exposure, some recorded events may have reflected nonspecific neurological worsening related to cancer, its treatment or comorbidity rather than new inflammatory MS activity, and recorded relapse rates may, therefore, have been overestimated. Taken together, these findings suggest relatively low recorded inflammatory activity early after cancer diagnosis in pwMS with recorded chemotherapy exposure, particularly in older individuals, and may provide cautious reassurance when interpreting individualised post-cancer DMT management within similar real-world care pathways. In contrast to relapse, CDP findings appeared more closely related to baseline neurological status than to post-cancer DMT category. Higher baseline disability and progressive phenotype were associated with CDP in the primary cohort, while CDP risk did not differ in matched analyses. Interpretation is limited by few events, incomplete EDSS follow-up and the possibility that disability worsening after cancer diagnosis reflected cancer morbidity, treatment effects, comorbidity or ascertainment patterns rather than MS progression.^9^

### Relation to existing evidence

Evidence on MS care after cancer remains limited. A US mixed-methods study (*n* = 43) of women with MS and breast cancer reported infrequent relapses and stable EDSS, with qualitative findings highlighting the uncertainty of managing a dual diagnosis and prioritisation of oncology care.^10^ A German cohort (*n* = 16) of pwMS with CNS tumours reported higher inflammatory activity in those who did not receive chemotherapy, with events clustering after the last documented lymphopenia.^11^ More recently, a multinational population-based cohort of pwMS with incident cancer (*n* = 6902) and matched cancer-free pwMS controls (*n* = 13,804) found reduced DMT use after cancer diagnosis, particularly among those receiving chemotherapy, without strong post-cancer change in neurologist visits or MS hospitalisations.^12^ These studies suggest that cancer diagnosis and treatment pathways commonly reshape MS prescribing, but evidence on subsequent relapse and disability outcomes remains limited. Our analysis adds international registry data on post-cancer DMT patterns together with relapse and 6-month CDP outcomes in pwMS with recorded chemotherapy exposure.

### Study limitations

This study has several limitations. It represents secondary use of MSBase data, which were not designed to capture cancer outcomes or oncological treatments in detail. The data set, therefore, cannot estimate cancer prevalence or report cancer-specific outcomes.

The cohort was heterogeneous, comprising multiple cancer types with differing biology, prognosis and treatment pathways. Attempts to validate chemotherapy regimens through site investigators were largely unsuccessful because cancer treatment often occurred outside neurology centres. Consequently, regimen data, chemotherapy timing, lymphocyte nadirs and immune recovery data were unavailable. Follow-up was, therefore, indexed to cancer diagnosis rather than chemotherapy completion, precluding separation of possible chemotherapy-related effects from immune recovery, DMT interruption or other aspects of cancer care. Findings should, therefore, be interpreted within the broader cancer care pathway. Although the breast cancer sensitivity analysis reduced heterogeneity, incomplete receptor status and histology data still limited treatment characterisation (Supplemental eTable 4). Other cancer treatments, including immune checkpoint inhibitors and hormonal therapies, were not captured and may also have influenced MS activity, introducing potential unmeasured confounding.^1,4,13^

The broad immunosuppressing versus non-immunosuppressing classification may also have obscured clinically relevant differences between specific DMTs or mechanism-based groups. Because post-cancer treatment selection was not random and was likely influenced by prior MS activity, disability, cancer characteristics, clinician preference and the broader cancer care pathway, comparisons between post-cancer DMT categories are susceptible to confounding by indication.

Early attrition after cancer diagnosis was itself a clinically relevant real-world finding, although the reasons for attrition could not be determined in this registry data set. Consistent with the US mixed-methods study noted above,^10^ this may in part reflect prioritisation of oncology care over MS during cancer treatment. These follow-up patterns may have biased ascertainment in more than one direction, with greater detection of neurological events and disability change in those under closer neurological review and under ascertainment in those less engaged with follow-up. The remaining cohort may, therefore, have overrepresented healthier survivors or those more engaged with MS care. Death after cancer diagnosis may also have acted as a competing risk for relapse or CDP.

Case ascertainment may also vary across countries. For example, MSBase operates as a national registry in the Czech Republic but is used mainly in specialised MS clinics in Australia, potentially influencing case mix. Finally, although analyses were limited to cancers diagnosed after 2010 to reflect the modern treatment era, the median diagnosis year of 2017 predates widespread use of newer DMTs, limiting generalisability to contemporary MS management.

## Conclusion

Among pwMS with recorded chemotherapy exposure, post-cancer DMT use shifted towards no recorded DMT and less immunosuppressing therapy by 12 months. Post-cancer DMT category was not associated with time to first relapse or 6-month CDP, while older age at cancer diagnosis was associated with lower relapse hazard. In matched analyses, relapse hazard was lower during the first year after cancer diagnosis only, although the drivers of this signal could not be separated from the broader cancer treatment pathway in this study design. These findings suggest relatively low recorded inflammatory activity, particularly in older individuals, and may cautiously inform interpretation of individualised post-cancer DMT management in similar clinical contexts. Prospective studies with regimen-level oncology data, treatment timing and immune recovery measures are needed.

## Supplemental Material

sj-docx-1-msj-10.1177_13524585261460658 – Supplemental material for Multiple sclerosis outcomes after cancer diagnosis in people with recorded chemotherapy exposure: A real-world MSBase study Supplemental material, sj-docx-1-msj-10.1177_13524585261460658 for Multiple sclerosis outcomes after cancer diagnosis in people with recorded chemotherapy exposure: A real-world MSBase study by Cassie Nesbitt, Paul Sanfilippo, Chao Zhu, Serkan Ozakbas, Alexandre Prat, Marc Girard, Pierre Duquette, Tomas Kalincik, Izanne Roos, Katherine Buzzard, Olga Skibina, Matteo Foschi, Andrea Surcinelli, Jeannette Lechner-Scott, Francesco Patti, Allan G Kermode, Marzena Fabis-Pedrini, William M Carroll, Suzanne Hodgkinson, Francois Grand’Maison, Eva Kubala Havrdova, Pavel Hradilek, Mario Habek, Davide Maimone, Raed Alroughani, Marta Vachova, Vincent van Pesch, Richard Macdonell, Daniele Spitaleri, Samia J Khoury, Oliver Gerlach, Koen de Gans, Pamela McCombe, Anneke Van Der Walt, Helmut Butzkueven and Vilija Jokubaitis in Multiple Sclerosis Journal

sj-docx-2-msj-10.1177_13524585261460658 – Supplemental material for Multiple sclerosis outcomes after cancer diagnosis in people with recorded chemotherapy exposure: A real-world MSBase study Supplemental material, sj-docx-2-msj-10.1177_13524585261460658 for Multiple sclerosis outcomes after cancer diagnosis in people with recorded chemotherapy exposure: A real-world MSBase study by Cassie Nesbitt, Paul Sanfilippo, Chao Zhu, Serkan Ozakbas, Alexandre Prat, Marc Girard, Pierre Duquette, Tomas Kalincik, Izanne Roos, Katherine Buzzard, Olga Skibina, Matteo Foschi, Andrea Surcinelli, Jeannette Lechner-Scott, Francesco Patti, Allan G Kermode, Marzena Fabis-Pedrini, William M Carroll, Suzanne Hodgkinson, Francois Grand’Maison, Eva Kubala Havrdova, Pavel Hradilek, Mario Habek, Davide Maimone, Raed Alroughani, Marta Vachova, Vincent van Pesch, Richard Macdonell, Daniele Spitaleri, Samia J Khoury, Oliver Gerlach, Koen de Gans, Pamela McCombe, Anneke Van Der Walt, Helmut Butzkueven and Vilija Jokubaitis in Multiple Sclerosis Journal

sj-docx-3-msj-10.1177_13524585261460658 – Supplemental material for Multiple sclerosis outcomes after cancer diagnosis in people with recorded chemotherapy exposure: A real-world MSBase study Supplemental material, sj-docx-3-msj-10.1177_13524585261460658 for Multiple sclerosis outcomes after cancer diagnosis in people with recorded chemotherapy exposure: A real-world MSBase study by Cassie Nesbitt, Paul Sanfilippo, Chao Zhu, Serkan Ozakbas, Alexandre Prat, Marc Girard, Pierre Duquette, Tomas Kalincik, Izanne Roos, Katherine Buzzard, Olga Skibina, Matteo Foschi, Andrea Surcinelli, Jeannette Lechner-Scott, Francesco Patti, Allan G Kermode, Marzena Fabis-Pedrini, William M Carroll, Suzanne Hodgkinson, Francois Grand’Maison, Eva Kubala Havrdova, Pavel Hradilek, Mario Habek, Davide Maimone, Raed Alroughani, Marta Vachova, Vincent van Pesch, Richard Macdonell, Daniele Spitaleri, Samia J Khoury, Oliver Gerlach, Koen de Gans, Pamela McCombe, Anneke Van Der Walt, Helmut Butzkueven and Vilija Jokubaitis in Multiple Sclerosis Journal

sj-docx-4-msj-10.1177_13524585261460658 – Supplemental material for Multiple sclerosis outcomes after cancer diagnosis in people with recorded chemotherapy exposure: A real-world MSBase study Supplemental material, sj-docx-4-msj-10.1177_13524585261460658 for Multiple sclerosis outcomes after cancer diagnosis in people with recorded chemotherapy exposure: A real-world MSBase study by Cassie Nesbitt, Paul Sanfilippo, Chao Zhu, Serkan Ozakbas, Alexandre Prat, Marc Girard, Pierre Duquette, Tomas Kalincik, Izanne Roos, Katherine Buzzard, Olga Skibina, Matteo Foschi, Andrea Surcinelli, Jeannette Lechner-Scott, Francesco Patti, Allan G Kermode, Marzena Fabis-Pedrini, William M Carroll, Suzanne Hodgkinson, Francois Grand’Maison, Eva Kubala Havrdova, Pavel Hradilek, Mario Habek, Davide Maimone, Raed Alroughani, Marta Vachova, Vincent van Pesch, Richard Macdonell, Daniele Spitaleri, Samia J Khoury, Oliver Gerlach, Koen de Gans, Pamela McCombe, Anneke Van Der Walt, Helmut Butzkueven and Vilija Jokubaitis in Multiple Sclerosis Journal

sj-docx-5-msj-10.1177_13524585261460658 – Supplemental material for Multiple sclerosis outcomes after cancer diagnosis in people with recorded chemotherapy exposure: A real-world MSBase study Supplemental material, sj-docx-5-msj-10.1177_13524585261460658 for Multiple sclerosis outcomes after cancer diagnosis in people with recorded chemotherapy exposure: A real-world MSBase study by Cassie Nesbitt, Paul Sanfilippo, Chao Zhu, Serkan Ozakbas, Alexandre Prat, Marc Girard, Pierre Duquette, Tomas Kalincik, Izanne Roos, Katherine Buzzard, Olga Skibina, Matteo Foschi, Andrea Surcinelli, Jeannette Lechner-Scott, Francesco Patti, Allan G Kermode, Marzena Fabis-Pedrini, William M Carroll, Suzanne Hodgkinson, Francois Grand’Maison, Eva Kubala Havrdova, Pavel Hradilek, Mario Habek, Davide Maimone, Raed Alroughani, Marta Vachova, Vincent van Pesch, Richard Macdonell, Daniele Spitaleri, Samia J Khoury, Oliver Gerlach, Koen de Gans, Pamela McCombe, Anneke Van Der Walt, Helmut Butzkueven and Vilija Jokubaitis in Multiple Sclerosis Journal

sj-docx-6-msj-10.1177_13524585261460658 – Supplemental material for Multiple sclerosis outcomes after cancer diagnosis in people with recorded chemotherapy exposure: A real-world MSBase study Supplemental material, sj-docx-6-msj-10.1177_13524585261460658 for Multiple sclerosis outcomes after cancer diagnosis in people with recorded chemotherapy exposure: A real-world MSBase study by Cassie Nesbitt, Paul Sanfilippo, Chao Zhu, Serkan Ozakbas, Alexandre Prat, Marc Girard, Pierre Duquette, Tomas Kalincik, Izanne Roos, Katherine Buzzard, Olga Skibina, Matteo Foschi, Andrea Surcinelli, Jeannette Lechner-Scott, Francesco Patti, Allan G Kermode, Marzena Fabis-Pedrini, William M Carroll, Suzanne Hodgkinson, Francois Grand’Maison, Eva Kubala Havrdova, Pavel Hradilek, Mario Habek, Davide Maimone, Raed Alroughani, Marta Vachova, Vincent van Pesch, Richard Macdonell, Daniele Spitaleri, Samia J Khoury, Oliver Gerlach, Koen de Gans, Pamela McCombe, Anneke Van Der Walt, Helmut Butzkueven and Vilija Jokubaitis in Multiple Sclerosis Journal
