# Supplementary material for: Multiple sclerosis outcomes after cancer diagnosis in people with recorded chemotherapy exposure: A real-world MSBase study
Source: Mult Scler. 2026 Aug 2;32(10):1083–97. doi: 10.1177/13524585261460658 (PMC13538901; doi:10.1177/13524585261460658)
Supplement: Supplementary file 1 [file sj-docx-1-msj-10.1177_13524585261460658.docx]

**e-Table 1 – DMT at 12 months after cancer diagnosis**

| **DMT at cancer diagnosis** | | | | | |
| --- | --- | --- | --- | --- | --- |
| Alemtuzumab 1 | Avonex 13 | Betaferon 12 | BTKI 1 | Anti-CD-20 combined 13 | |
| Cladribine 2 | Cyclophosphamide 1 | Dimethyl fumarate 8 | Fingolimod 41 | Glatiramer acetate 30 | |
| Natalizumab 16 | Non-specific 18 | Plegridy 4 | Rebif 22 | Teriflunomide 8 | |
| Siponimod 3 | No DMT 106 | | | | |
| **DMT 1 year after cancer diagnosis** | | | | | |
| Alemtuzumab 1 | AHSCT 1 | Avonex 10 | Betaferon 9 | Anti-CD-20 combined 12 | |
| Cladribine 3 | Cyclophosphamide 3 | Dimethyl fumarate 5 | Fingolimod 15 | Glatiramer acetate 46 | |
| Natalizumab 6 | Non-specific 12 | Plegridy 1 | Rebif 18 | Siponimod 1 | |
| Teriflunomide 9 | - | | | | |
| Among those with no DMT at 1 year | | | | | |
| No DMT before or after cancer 45 | | | | | |
| Discontinued before cancer diagnosis and remained off treatment 45 | | | | | |
| Discontinued within six months after cancer diagnosis and remained off treatment 52 | | | | | |
| Initiated first DMT more than two years after cancer 5 | | | | | |
| **DMT group differences 12 months after cancer diagnosis** | | | | | |
| Variable | | | | Diff  p= | Post hoc |
| DMT | Immunosuppressing | Non-  Immunosuppressing | No DMT |  |  |
| n 299 | 59 (19.7%) | 93 (31.1%) | 147 (49.2%) |  |  |
| Sex | F 47, M 12 | F 76 M 17 | F 114 M 33 | 0.7 | - |
| Age at cancer | 54.7 years  (IQR 16.5) | 49.9 years  (IQR 12.6) | 53 years  (IQR 15.7) | 0.01 | N-IS < None p 0.007 |
| MS duration at cancer | 13.9 years  (IQR 11.9) | 12 years  (IQR 12.1) | 13.9 years  (IQR 16.3) | 0.38 | - |
| First EDSS | 3 med (IQR 2) | 2 med (IQR 1.5) | 2.5 med (IQR 2.5) | 0.05 | IS>N-IS p= 0.03 |
| EDSS at cancer | 3 med (IQR 4) | 2.5 med (IQR 2.5) | 2.5 med (IQR 3.9) | 0.4 | - |
| Missing EDSS between groups First EDSS p 0.17 Cancer EDSS p=0.33 | | | | | |
| Cancer Type | | | | | |
| Adreno-urinary | 2 | 1 | 2 | - | - |
| Anal | 1 | - | 1 |  |  |
| Blood | 13 | 5 | 20 |  |  |
| Brain and nerve | 1 | - | 1 |  |  |
| Breast | 25 | 47 | 71 |  |  |
| CNS lymphoma | 1 | - | - |  |  |
| Cervix | 1 | 3 | 1 |  |  |
| Colorectal | 3 | 9 | 25 |  |  |
| Gastro-oesophageal | - | 2 | 3 |  |  |
| Head and neck | 1 | 2 | 3 |  |  |
| Hepatobiliary | 1 | 1 | 2 |  |  |
| Lung | 2 | 2 | 3 |  |  |
| Melanoma | 1 | 2 | 3 |  |  |
| Mesothelioma | - |  | 2 |  |  |
| Neuro-endocrine | - | 1 | - |  |  |
| Ovarian | 2 | 6 | 1 |  |  |
| Pancreas | - | - | 3 |  |  |
| Prostate | - | 2 | 2 |  |  |
| Sarcoma | 1 | 1 | - |  |  |
| Skin | - | 1 | 1 |  |  |
| Testicular | 1 | 4 | - |  |  |
| Unknown primary | - | 1 | - |  |  |
| Uterus | 3 | 1 | 1 |  |  |
| Vulvar-vaginal | - | 1 | - |  |  |

med median; IQR = interquartile range; IS = immunosuppressing DMTs; NIS = non-immunosuppressing DMTs, Diff = group differences, nonspecific immunosuppression = mycophenolate, azathioprine, methotrexate, BTKI = Bruton tyrosine kinase inhibitor, Anti-CD-20 combined = Rituximab, Ocrelizumab
