# Supplementary material for: Multiple sclerosis outcomes after cancer diagnosis in people with recorded chemotherapy exposure: A real-world MSBase study
Source: Mult Scler. 2026 Aug 2;32(10):1083–97. doi: 10.1177/13524585261460658 (PMC13538901; doi:10.1177/13524585261460658)
Supplement: Supplementary file 2 [file sj-docx-2-msj-10.1177_13524585261460658.docx]

eTable 2 - Disease-modifying therapy class transitions from cancer diagnosis to 12 months after diagnosis

| **DMT at cancer diagnosis** | **Immunosuppressing at 12 months** | **Non-immunosuppressing at 12 months** | **No DMT at 12 months** | **Total** |
| --- | --- | --- | --- | --- |
| **Immunosuppressing** | **41 (39.4%)** | **18 (17.3%)** | **45 (43.3%)** | **104** |
| **Non-immunosuppressing** | **0 (0.0%)** | **69 (77.5%)** | **20 (22.5%)** | **89** |
| **No recorded DMT** | **18 (17.0%)** | **6 (5.7%)** | **82 (77.4%)** | **106** |
| **Total** | **59** | **93** | **147** | **299** |
