# Supplementary material for: Multiple sclerosis outcomes after cancer diagnosis in people with recorded chemotherapy exposure: A real-world MSBase study
Source: Mult Scler. 2026 Aug 2;32(10):1083–97. doi: 10.1177/13524585261460658 (PMC13538901; doi:10.1177/13524585261460658)
Supplement: Supplementary file 3 [file sj-docx-3-msj-10.1177_13524585261460658.docx]

**eTABLE 3: Covariate balance before and after propensity score matching**

| **Variable** | **Pre-Match** | | | **Post-match** | | |
| --- | --- | --- | --- | --- | --- | --- |
|  | **Cancer + Chemo** | **Controls** | **Pre-match SMD** | **Cancer + Chemo** | **Controls** | **Post-match SMD** |
| Age at index visit (years) | 52.3 | 42.1 | **0.95** | 52.3 | 52.3 | 0.00 |
| Visit calendar year | 2017.0 | 2014.6 | **0.69** | 2017.0 | 2017.0 | 0.00 |
| Age at MS onset (years) | 33.0 | 34.4 | 0.14 | 33.0 | 31.9 | 0.11 |
| Disease duration (years) | 14.5 | 10.7 | **0.39** | 14.5 | 15.6 | 0.11 |
| EDSS at index | 3.13 | 2.60 | **0.25** | 3.21 | 3.50 | **0.19** |
| Relapse count | 0.14 | 0.41 | **0.65** | 0.14 | 0.12 | 0.07 |
| Female sex (%) | 79.8 | 71.8 | **0.20** | 79.3 | 80.5 | 0.03 |
| **Prior DMT exposure at index (cancer diagnosis of matched visit)** | | | | | | |
| Interferons (combined) | 61.1 | 62.7 | 0.02 | 60.9 | 61.1 | 0.00 |
| Glatiramer acetate | 26.3 | 20.7 | 0.13 | 26.2 | 24.0 | 0.05 |
| Teriflunomide | 5.0 | 6.1 | 0.05 | 5.1 | 6.3 | 0.05 |
| Fumarates (combined) | 5.3 | 6.7 | 0.06 | 5.5 | 7.0 | 0.07 |
| Natalizumab | 12.2 | 12.2 | 0.00 | 12.1 | 11.3 | 0.02 |
| S1P modulators (combined) | 19.9 | 14.0 | 0.14 | 18.0 | 18.0 | 0.00 |
| Anti-CD20 (combined) | 5.7 | 6.0 | 0.01 | 5.5 | 4.3 | 0.05 |
| Immune reconstitution therapy (IRT) | 3.4 | 3.0 | 0.02 | 3.5 | 5.5 | 0.11 |
| Non-specific immunosuppression | 11.1 | 10.1 | 0.03 | 11.3 | 11.9 | 0.02 |
| BTK inhibitors | 0.0 | <0.1 | 0.01 | 0.0 | 0.0 | 0.00 |
| Daclizumab | 0.8 | 0.2 | 0.07 | 0.0 | 0.0 | 0.00 |
| **Current DMT at index (cancer diagnosis of matched visit)** | | | | | | |
| No DMT | 44.3 | 29.4 | **0.30** | 43.0 | 41.6 | 0.03 |
| Interferon | 14.1 | 26.0 | **0.34** | 14.5 | 15.8 | 0.04 |
| Glatiramer acetate | 12.6 | 9.4 | 0.10 | 12.9 | 10.9 | 0.06 |
| Teriflunomide | 3.1 | 4.1 | 0.06 | 3.1 | 2.9 | 0.01 |
| Fumarates | 2.3 | 4.1 | 0.12 | 2.3 | 4.1 | 0.12 |
| S1P modulators | 10.3 | 9.5 | 0.03 | 10.5 | 9.4 | 0.04 |
| Natalizumab | 5.0 | 7.9 | 0.14 | 5.1 | 5.3 | 0.01 |
| Anti-CD20 | 2.3 | 5.1 | **0.19** | 2.3 | 3.1 | 0.05 |
| IRT | 0.8 | 0.8 | 0.01 | 0.8 | 1.2 | 0.04 |
| Non-specific immunosuppression | 4.2 | 3.2 | 0.05 | 4.3 | 3.9 | 0.02 |

Comparison of Outcomes Before and After Propensity Score Matching. Balance was targeted at SMD <0.15.^13^ Variables with SMD >0.15 are highlighted in bold.

Interferons combined = Interferon beta-1a, Interferon beta-1b, Pegylated Interferon beta-1, Interferon therapy not otherwise specified, Combined fumarates = Dimethyl fumarate + Diroximel fumarate, Combined S1P Fingolimod, Siponimod, Ozanimod, Ponesimod, BTKI Bruton’s tyrosine kinase inhibitors, anti-CD20 = Rituximab + Ocrelizumab + Ofatumumab, Combined IRT = Cladribine + Alemtuzumab, Non-Specific immunosuppression = mycophenolate, methotrexate, azathioprine), Relapse count = number of relapses recorded in the 12 months prior to cancer diagnosis/matched visit date
