# Supplementary material for: Multiple sclerosis outcomes after cancer diagnosis in people with recorded chemotherapy exposure: A real-world MSBase study
Source: Mult Scler. 2026 Aug 2;32(10):1083–97. doi: 10.1177/13524585261460658 (PMC13538901; doi:10.1177/13524585261460658)
Supplement: Supplementary file 4 [file sj-docx-4-msj-10.1177_13524585261460658.docx]

**eTable 4: Breast Cancer Analysis**

| n= | 172 | |
| --- | --- | --- |
| Sex* | Female 99.4% | |
|  | Male 0.6% | |
| Geographical distribution | Argentina: 0.58% | Ireland: 2.33% |
|  | Australia: 25.58% | Italy: 11.63% |
|  | Belgium: 1.74% | Kuwait: 2.33% |
|  | Brazil: 0.58% | Lebanon: 1.16% |
|  | Canada: 11.05% | Netherlands: 4.65% |
|  | Colombia: 0.58% | Portugal: 1.16% |
|  | Czech Republic: 14.53% | Romania: 0.58% |
|  | Egypt: 0.58% | Saudi Arabia: 0.58% |
|  | Spain: 4.65% | Slovenia: 0.58% |
|  | United Kingdom: 1.16% | Turkey: 14.53% |
|  | Croatia: 2.91% | |
| **MS characteristics** | | |
| Median age at MS onset years (IQR) | 35.94 (13.62) | |
| Median follow up years | 3.9 years (after cancer) | |
| MS phenotype at cancer diagnosis | Clinically Isolated syndrome 8.85% | |
|  | Primary Progressive 3.65% | |
|  | Progressive Relapsing: 2.08% | |
|  | Relapse Onset “RRMS”: 73.44%, | |
|  | Secondary Progressive: 11.98% | |
| Disability scores (EDSS) | | |
| Timepoint (+/- 6months) | Median (IQR) | Missing (%) |
| Baseline (at cancer) | 2.5 (3.25) | 48.4% |
| 12 months | 2.5(3.5) | 66.91% |
| 24 months | 2.5(3.5) | 54.1% |
| 36 months | 2.25(3.5) | 62.8% |
| **Cancer characteristics** | | |
| Median age at cancer onset years | 48.8 (13.71) | |
| Histology (when available) | | |
| Invasive Ductal | 35 | |
| Invasive Lobular | 2 | |
| DCIS | 1 | |
| Rare | 3 papillary, 1 tubular, 1 mucinous | |
| Mixed | 1 | |
| Receptor status (where available) | | |
| ER (Estrogen Receptor) + (positive) | 1 | |
| Luminal A (ER+ PR+ HER2-) | 6 | |
| Luminal B (ER+ PR+ HER2 +) | 7 | |
| HER 2+ ER/PR - | 1 | |
| Triple negative | 3 | |
| Metastatic | 3 | |
| Recorded cancer outcomes (where available) | | |
| Cancer related deaths | 3 | |
| Ongoing | 58 | |
| Remission | 78 | |
| Other | 5 | |
| Recurrence/second cancer | 5 | |
