# Supplementary material for: Multiple sclerosis outcomes after cancer diagnosis in people with recorded chemotherapy exposure: A real-world MSBase study
Source: Mult Scler. 2026 Aug 2;32(10):1083–97. doi: 10.1177/13524585261460658 (PMC13538901; doi:10.1177/13524585261460658)
Supplement: Supplementary file 5 [file sj-docx-5-msj-10.1177_13524585261460658.docx]

**eTable 5: Covariate balance before and after propensity score matching**

| **Variable** | **Pre-Match** | | | **Post-match** | | |
| --- | --- | --- | --- | --- | --- | --- |
|  | **Cancer + Chemo** | **Controls** | **SMD** | **Cancer + Chemo** | **Controls** | **SMD** |
| Age at index visit (rounded) | 50.44 | 42.08 | **0.81** | 50.38 | 50.38 | 0.00 |
| Calendar year of visit | 2016.62 | 2014.64 | **0.56** | 2016.51 | 2016.51 | 0.00 |
| Relapse count | 0.20 | 0.41 | **0.41** | 0.21 | 0.18 | 0.06 |
| EDSS | 2.81 | 2.60 | 0.11 | 2.80 | 2.94 | 0.07 |
| Age at MS onset (years) | 33.24 | 34.43 | 0.13 | 33.00 | 33.08 | 0.01 |
| MS disease duration (years) | 13.84 | 10.70 | **0.35** | 13.99 | 13.92 | 0.01 |
| Female sex | 1.00 | 0.72 | **0.63** | 1.00 | 1.00 | 0.00 |
| Prior DMT exposure at index (cancer diagnosis of matched visit) | | | | | | |
| Interferons (combined) | 0.57 | 0.63 | 0.08 | 0.58 | 0.65 | 0.11 |
| Glatiramer acetate | 0.28 | 0.21 | **0.17** | 0.28 | 0.32 | 0.09 |
| Teriflunomide | 0.07 | 0.06 | 0.05 | 0.07 | 0.10 | 0.13 |
| Fumarates (combined) | 0.05 | 0.07 | 0.09 | 0.05 | 0.04 | 0.06 |
| S1P modulators (combined) | 0.16 | 0.14 | 0.06 | 0.15 | 0.15 | 0.01 |
| Natalizumab | 0.15 | 0.12 | 0.09 | 0.14 | 0.14 | 0.01 |
| Anti-CD20 therapies | 0.05 | 0.06 | 0.05 | 0.03 | 0.03 | 0.00 |
| Immune reconstitution therapies (IRT) | 0.04 | 0.03 | 0.05 | 0.04 | 0.05 | 0.02 |
| BTK inhibitors | 0.00 | 0.00 | 0.01 | 0.00 | 0.00 | 0.00 |
| Daclizumab | 0.01 | 0.00 | 0.07 | 0.00 | 0.00 | 0.00 |
| Non-specific immunosuppression | 0.10 | 0.10 | 0.01 | 0.11 | 0.12 | 0.04 |
| Current DMT at index (cancer diagnosis of matched visit) | | | | | | |
| No DMT | 0.44 | 0.29 | **0.29** | 0.42 | 0.36 | 0.10 |
| Interferon | 0.15 | 0.26 | **0.33** | 0.15 | 0.19 | 0.12 |
| Glatiramer acetate | 0.13 | 0.09 | 0.10 | 0.13 | 0.14 | 0.01 |
| Teriflunomide | 0.04 | 0.04 | 0.00 | 0.04 | 0.08 | 0.09 |
| Fumarates | 0.02 | 0.04 | 0.11 | 0.03 | 0.01 | 0.08 |
| S1P modulators | 0.07 | 0.10 | 0.09 | 0.08 | 0.08 | 0.02 |
| Natalizumab | 0.08 | 0.08 | 0.01 | 0.08 | 0.07 | 0.06 |
| Anti-CD20 | 0.01 | 0.05 | **0.48** | 0.01 | 0.01 | 0.00 |
| IRT | 0.02 | 0.01 | 0.06 | 0.02 | 0.02 | 0.00 |
| Non-specific immunosuppression | 0.05 | 0.03 | 0.08 | 0.05 | 0.05 | 0.00 |

Comparison of Outcomes Before and After Propensity Score Matching. Balance was targeted at SMD <0.15.^13^ Variables with SMD >0.15 are highlighted in bold.

Interferons combined = Interferon beta-1a, Interferon beta-1b, Pegylated Interferon beta-1, Interferon therapy not otherwise specified, Combined fumarates = Dimethyl fumarate + Diroximel fumarate, Combined S1P Fingolimod, Siponimod, Ozanimod, Ponesimod, BTKI Bruton’s tyrosine kinase inhibitors, anti-CD20 = Rituximab + Ocrelizumab + Ofatumumab, Combined IRT = Cladribine + Alemtuzumab, Non-Specific immunosuppression = mycophenolate, methotrexate, azathioprine), Relapse count = number of relapses recorded in the 12 months prior to cancer diagnosis/matched visit date
