# Supplementary material for: Multiple sclerosis outcomes after cancer diagnosis in people with recorded chemotherapy exposure: A real-world MSBase study
Source: Mult Scler. 2026 Aug 2;32(10):1083–97. doi: 10.1177/13524585261460658 (PMC13538901; doi:10.1177/13524585261460658)
Supplement: Supplementary file 6 [file sj-docx-6-msj-10.1177_13524585261460658.docx]

**eTable 6: Young patient sub-group Analysis**

| **Characteristic** | **Value** |
| --- | --- |
| **N= 55** | |
| **Sex* %** | Female 76.9% |
|  | Male 23.1% |
| **MS characteristics** | |
| **Disease duration at time of cancer** | Median 8.02 years (IQR: 8.69) |
| **MS phenotype at cancer diagnosis** | Clinically isolated syndrome n=5 |
|  | Relapse onset n=43 |
|  | Secondary Progressive n=4 |
| Disability outcomes (EDSS) | |
| **Timepoint** | Median (IQR) |
| **Baseline at cancer +/-6months** | 2 (1.5) |
| **12-18 months post-cancer** | 2 (2.5) |
| **18 to 30 months post-cancer** | 2 (2.25) |
| **30 to 42 months post-cancer** | 1.5 (2.25) |
| **DMT at cancer diagnosis (n=)** | Alemtuzumab: 1 |
|  | Avonex: 4 |
|  | Azathioprine: 4 |
|  | Fumarates: 1 |
|  | Fingolimod: 3 |
|  | Glatiramer: 8 |
|  | Methotrexate: 1 |
|  | Natalizumab: 6 |
|  | None: 17 |
|  | Ocrelizumab: 2 |
|  | Plegridy: 2 |
|  | Rebif: 6 |
| **DMT decisions 12 months after cancer** | None n=26 |
|  | Same n=14 |
|  | Other n=15 |
| **Cancer Characteristics** | |
| **Median age at cancer diagnosis (IQR)** | 36.3 (5.7) |
| **Cancer mortality** | n=1 |
| **Cancer type (n=)** | Blood 8 |
|  | Brain + Nerve 1 |
|  | Breast 24 |
|  | CNS lymphoma 1 |
|  | Colo-rectal 4 |
|  | Head + Neck 3 |
|  | Melanoma 1 |
|  | Neuroendocrine 1 |
|  | Ovarian 2 |
|  | Sarcoma 2 |
|  | Skin 1 |
|  | Testicular 3 |
|  | Thyroid 1 |
